# Non–steady state thermometry with optical diffraction tomography

**DOI:** 10.1126/sciadv.adk5440

**Published:** 2024-03-22

**Authors:** Adarsh B. Vasista, Bernard Ciraulo, Falko Schmidt, Jaime Ortega Arroyo, Romain Quidant

**Affiliations:** ^1^ Nanophotonic Systems Laboratory, Department of Mechanical and Process Engineering, ETH-Zürich, 8092 Zürich, Switzerland.; ^2^ Pediatric Molecular Neuro-Oncology Research, University Children’s Hospital Zürich, Balgrist Campus, 8008 Zürich, Switzerland.

## Abstract

Label-free thermometry is a pivotal tool for many disciplines. However, most current approaches are only suitable for planar heat sources in steady state, thereby restricting the range of systems that can be reliably studied. Here, we introduce pump probe–based optical diffraction tomography (ODT) as a method to map temperature precisely and accurately in three dimensions (3D) at the single-particle level. To do so, we first systematically characterize the thermal landscape in a model system consisting of gold nanorods in a microchamber and then benchmark the results against simulations and quantitative phase imaging thermometry. We then apply ODT thermometry to resolve thermal landscapes inaccessible to other label-free approaches in the form of nonplanar heat sources embedded in complex environments and freely diffusing gold nanorods in a microchamber. Last, we foresee that our approach will find many applications where routine thermal characterization of heterogeneous nanoparticles samples in 3D or in non–steady state is required.

## Teaser

We present a label-free optical approach that spatially maps temperature from 3D heat sources at the single-particle level.

## INTRODUCTION

Measuring temperature reliably at the nano- and microscale is not only key to answering fundamental thermodynamic questions at these scales but also in a variety of applications such as photothermal cancer therapy (*1*–*3*), drug delivery (*4*), photocatalysis (*5*, *6*), thermal lensing (*7*, *8*), microfluidics (*9*–*12*), vibrational spectroscopy using mid-infrared photothermal microscopy (*13*–*15*), etc. Nonetheless, the nonpropagative nature of heat poses a challenge to reliably and accurately measure temperature at these scales, especially under non–steady state conditions.

Various optical thermometry techniques have recently emerged to address this need, and we can broadly categorize them into label-based and label-free methods. The working principle for label-based methods relies on measuring a temperature sensitive emission signature such as Raman scattering (*16*, *17*), fluorescence anisotropy (*18*), fluorescence intensity (*19*, *20*), fluorescence spectra (*21*, *22*), and photoluminescence life time (*23*) from a set of molecular probes. While these methods can measure temperature under non–steady state conditions, they face drawbacks such as slow readout rates (*21*), low sensitivity (*24*), lack of reliability (*19*, *20*), and the need to place the molecular probes in the system, which is not always feasible. To circumvent these issues, label-free methods such as infrared imaging (*25*), x-ray absorption spectroscopy (*26*), quantitative phase imaging (QPI) (*27*), and mid-infrared photothermal microscopy (*13*–*15*) have been proposed. Of all label-free approaches, QPI is one of the most promising ones due to its ease of implementation into commercial microscopes, its speed, and high-resolution temperature retrieval.

QPI thermometry is based on measuring the optical path differences of a probe beam as a result of the small temperature-induced changes in the refractive index of a material. Several different implementations of QPI exist, either in the form of inline (*28*), off-axis (*9*, *12*), or shearing-based holography (*27*, *29*, *30*), yet all extract an optical path length difference from the measured phase change. Nonetheless, retrieving temperature profiles from these optical path length changes relies on algorithms that assume that the system is in steady state, all the heat sources lie in the same plane, and the temperature profile follows a $\frac{1}{r}$ decay from the heat source, where *r* is the radial coordinate (*27*). If any one of these conditions fail, then deriving a suitable temperature retrieval model becomes increasingly difficult, if not impractical or intractable. Unfortunately, these assumptions restrict the range of systems to which QPI thermometry can be applied.

As a promising alternative to QPI thermometry, optical diffraction tomography (ODT) has been widely used to accurately determine three-dimensional (3D) refractive index maps of biological systems (*31*–*37*), to study material anisotropy (*38*), and to perform vibrational spectroscopy based on mid-infrared photothermal microscopy (*14*). Recently, ODT-based thermometry has been experimentally demonstrated in the steady state (*39*) without the need of any assumptions other than a look-up table that relates the measured 3D refractive index change from a thermo-optical material to a temperature change. However, the impact and applicability of the ODT technique to investigate a wide range of heat transfer problems containing nonplanar heat sources under non–steady state conditions, which are inaccessible to QPI, are yet to be established.

In this work, we systematically studied, experimentally and numerically, a non–steady state system in the form of a microchamber undergoing photothermal conversion by gold nanorods (AuNRs) to assess the performance of ODT-based thermometry. We specifically use QPI to benchmark the conditions that push the system away from steady state and to identify the mechanism responsible for such. We find that for our system, heat accumulation extends the time to reach steady state and can be tuned by either the height of the microchamber or the thermal conductivity of the surroundings. Under non–steady state conditions, we validate ODT thermometry against simulations and showcase that QPI, despite accurately retrieving the temperature gradient, underestimates the absolute value of the local temperature. Last, we apply ODT thermometry to three representative non–steady state systems, where photothermal conversion is achieved by nonplanar heat sources in the form of the following: colloidal gold nanoparticles freely diffusing in aqueous media, gold nanoshells (AuNS) embedded in a 3D hydrogel, and of AuNRs internalized by cells. Hence, our work highlights a promising approach to addressing the knowledge gap of heat propagation and thermometry at the nano- and microlength scales.

## RESULTS

### Working principle

**Fig. 1.**
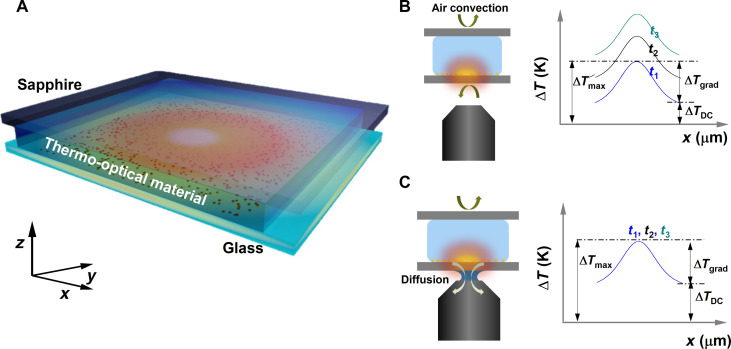
The sample and the technique. (**A**) Schematic representation of the microchamber sample formed by a AuNR-functionalized glass substrate and sapphire superstrate separated by a silicone spacer. The chamber is filled with water as the thermo-optical material with a known refractive index. A 785-nm laser beam resonantly excites the AuNRs, causing the medium surrounding the AuNRs to heat up and change its refractive index. A probe laser at 465 nm measures the phase shift due to the altered refractive index. (**B** and **C**) Schematics representing the phenomena of heat transfer in the microchamber when the chamber is probed using air-immersion and oil-immersion objectives, respectively, and its effect on the temporal evolution of the thermal profiles. In the case of the air objective, the primary mechanism of heat transfer is natural air convection resulting in the continuous increase of the thermal floor (Δ*T*_DC_) unlike the oil-immersion case where the immersion oil acts as a thermal bridge between the chamber and the metallic case of the objective lens.

Figure 1 depicts the working principle of the experiment. As nano sources of heat, we used AuNRs immobilized on a glass substrate (see Methods). A microfluidic chamber was, then, prepared by sandwiching a thermo-optical material, water in our case, between the glass substrate and a sapphire superstrate (Fig. 1A). A pump beam of wavelength close to the absorption maximum of the AuNRs (785 nm) excites the substrate and heats the sample. This, in turn, changes the temperature-dependent refractive index of water, thereby spatially encoding the thermal profile in the form of wavefront changes. Measuring these temperature-induced wavefront changes forms the basis of either phase-based temperature techniques: QPI and ODT thermometry.

When a pump laser heats the AuNRs, we can define two characteristic timescales defining the thermodynamic state of the system. (i) The timescale to reach the local steady state in the immediate vicinity of the heated nanorods. This timescale can be understood as the time for the thermal gradient in the sample to establish and is of the order of a few milliseconds for a typical beam size of ~10 μm. (ii) The timescale for the entire microchamber system to reach steady state. This parameter is a complex function of the volume of the thermo-optical material, translating to the chamber height, and the thermal conductivity of the surroundings.

If the microchamber is not in thermal contact with a heat sink, then the chamber thermalizes with the outside environment through natural air convection, as shown in Fig. 1B. In this case, local thermalization happens quickly, setting up the thermal gradient. However, the lower efficiency of the natural air convection limits the dynamics of the heat transfer and results in the build-up of heat inside the microchamber. This heat build-up in the chamber manifests as a constant increase in the temperature profile. If one monitors the temporal evolution of the thermal profile, although the microchamber reaches a local steady state quickly, establishing the thermal gradient (Δ*T*_grad_), it experiences a continuous increase in the thermal floor (Δ*T*_DC_).

The thermodynamics of the heated microchamber can be markedly altered by providing access to a thermal sink by substituting the air-immersion objective with an oil-immersion one. In such a case, the refractive index matching oil acts as a thermal bridge between the microchamber and the metallic body of the objective lens and its respective optomechanical elements, as shown in Fig. 1C. As the microchamber is in thermal contact with a large thermal sink, the time taken to reach the global steady state reduces considerably, thus avoiding the continuous increase of the thermal floor (Δ*T*_DC_).

### Time evolution of phase maps

**Fig. 2.**
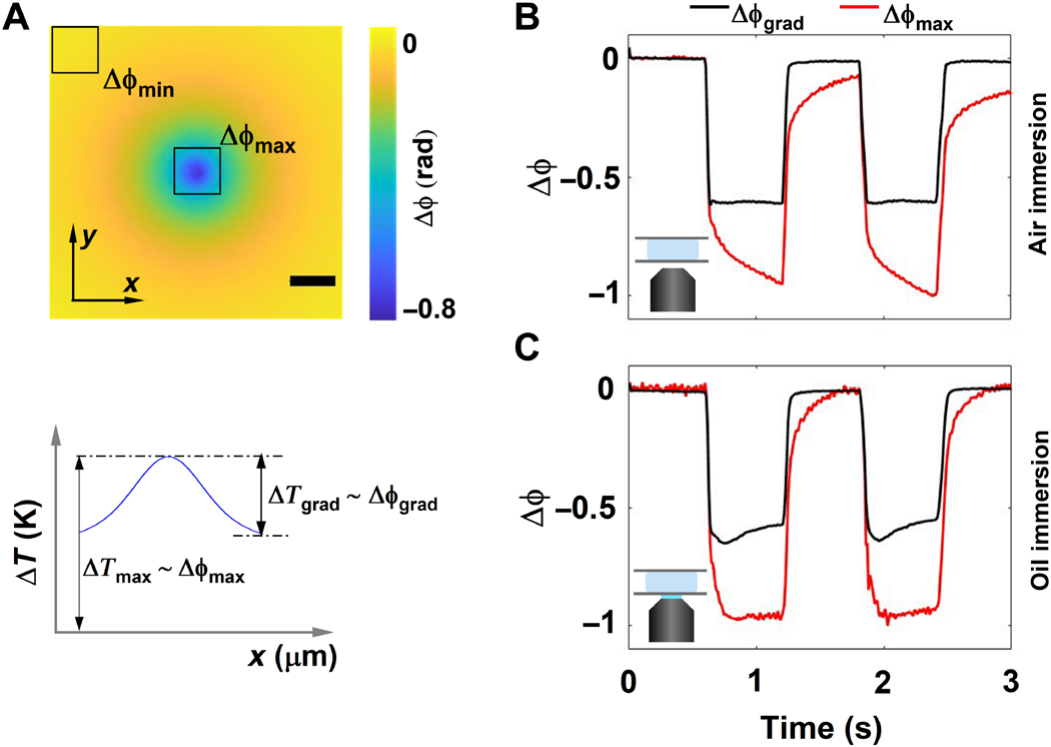
Assessing steady-state dynamics by phase imaging. (**A**) Representative phase difference image measured by subtracting the phase image with the heating laser switched ON from a reference phase image measured with heating laser switched OFF and its corresponding thermal profile properties (top and bottom, respectively). The maximum and minimum phase shifts within the field of view, acquired because of the heating of nanorods, are termed Δϕ_max_ and Δϕ_min_, respectively. The difference between the Δϕ_max_ and Δϕ_min_ determines the phase gradient (Δϕ_grad_) of the phase image. The maximum phase shift accumulated (Δϕ_max_) corresponds, in a closed system, to the absolute increase in the temperature (Δ*T*_max_), and the phase gradient (Δϕ_grad_) corresponds to the thermal gradient in the image (Δ*T*_grad_) Scale bar: 15 μm. (**B** and **C**) Time evolution of Δϕ_grad_ and Δϕ_max_ for a chamber height of 500 μm probed using air- and oil-immersion objectives respectively.

To understand the thermodynamics of the microchamber, we study the temporal evolution of phase difference maps with pump-probe phase imaging in an off-axis holography configuration (Fig. 2 and see Supplementary Methods). The AuNRs in the microchamber were heated upon irradiation with a time-modulated pump laser of wavelength 785 nm (close to the absorption maximum of the nanorods). The optical path difference due to optical pumping and thereby local heating was measured by the difference between pump ON and pump OFF states of a probe beam with a wavelength of 465 nm (*9*).

For quasi-infinite systems and at *r* = 0, the steady state occurs on a timescale given by $\tau ∼\frac{D^{2}}{4a_{s}}$ (*7*), where *D* is the diameter of the heat source and *as* is the thermal diffusivity of the medium. In the case of photothermal conversion of nanoparticle ensembles, i.e., this experiment, the diameter of the heat source corresponds to the size of the pump beam. Therefore, for a pump beam size of 10 μm, τ∼ 175 μs. However, this timescale does not hold for positions away from the center of the heat source nor for finite systems such as a microchamber. Instead, the time to reach steady state is a complex function of the height of the chamber, the thermal conductivities of the substrate and the superstrate, the position away from the heat source, and the heat transfer properties of the thermal sink.

To follow the thermal dynamics of the system, we tracked the temporal evolution of the phase of the probe beam, which is a suitable metric for finite real-world systems given the relation between optical path difference and temperature. Figure 2A shows a representative phase difference map between pump ON (hot) and pump OFF(cold) states, where a substantial phase dip at the center (laser excitation spot) is observed, as expected because of the negative thermo-optical coefficient of water. To understand the temporal phase response, we defined two important parameters: (i) Δϕ_max_, the maximum phase shift acquired by the probe beam due to heating; and (ii) Δϕ_grad_, the maximum phase gradient in the image calculated by subtracting the phase difference value at the edge of the phase image (Δϕ_min_) from the phase change induced at the center of the image due to heating. Intuitively, Δϕ_max_ and Δϕ_grad_ report on the absolute temperature change and thermal gradient in the microchamber, respectively, as represented by Fig. 2A. For instance at steady state, when the temperature no longer changes within the sample, we expect the phase to converge to a constant value.

To understand the temperature dynamics of the mircochamber as a function of the thermal conductivity of the surroundings, we followed the evolution of Δϕ_max_ and Δϕ_grad_ in a microchamber with a height of 500 μm probed in two different configurations: in the absence (air-immersion objective) and presence (oil-immersion objective) of a thermal sink (Fig. 2, B and C). In the absence of a thermal sink, measurements with an air objective (Fig. 2B), the dynamics of the Δϕ_max_ did not saturate within the timescale of the experiment, whereas Δϕ_grad_ saturated shortly after the pump pulse was switched ON. This indicates on the one hand that the system had not reached a steady state. On the other hand, although the system was not in steady state, the shape of the thermal profile remained the same, as indicated by the stabilization of the phase gradient.

Figure 2C shows the time evolution of Δϕ_max_ and Δϕ_grad_ of the microchamber in the presence of a thermal sink using an oil-immersion objective lens. Here, Δϕ_max_ saturated within the timescale of the experiment, while the Δϕ_grad_ did not. Specifically, Δϕ_grad_ showed time-dependent variations, indicating that the system is still evolving and that they are affected by the thermal conductivities of the surrounding environment (fig. S8). Comparing Fig. 2 (B and C), we can conclude that coupling the system to a heat sink via the oil immersion modifies the thermal dynamics by specifically pushing the system toward the steady state much faster compared to the air-immersion configuration.

**Fig. 3.**
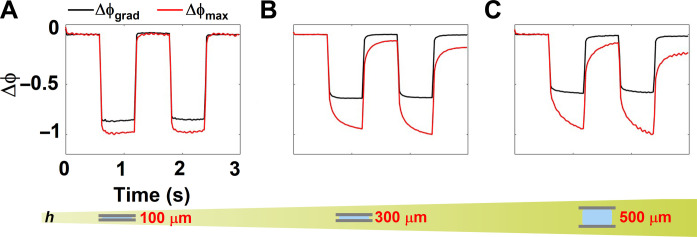
Assessing steady-state dynamics by phase imaging. (**A** to **C**) The time evolution of the phase gradient Δϕ_grad_ and maximum phase shift accumulated Δϕ_max_ for chamber heights of 100, 300, and 500 μm probed using an air objective, respectively.

Further, to understand the effect of height of the microchamber on the temperature dynamics, we studied the temporal evolution of the phase for three different chamber heights at a fixed pump beam size and power in the air-immersion configuration (Fig. 3, A to C). For the 100-μm chamber, the dynamics of Δϕ_max_ and Δϕ_grad_ saturated within the timescales of the experiment, suggesting that the system had reached steady state within the pulse duration of the pump beam (600 ms). Upon increasing the chamber height to either 300 or 500 μm, Δϕ_max_ no longer saturated, whereas Δϕ_grad_ did so shortly after the pump pulse was switched ON. As the height of the microchamber was increased, so did the distance between the heat source and the high–thermal conductivity sapphire superstrate, which acts a heat sink, thus affecting the thermodynamics of the chamber. These results, again, indicate that the system with increased chamber heights had not reached steady state; however, the shape of the temperature profile remained the same, as indicated by the stabilization of the thermal gradient. That is, the difference between the temperatures probed by Δϕ_max_ and Δϕ_grad_ corresponded to a uniform temperature shift, DC offset, within the imaged area. Note that the thermal relaxation dynamics (cooling) also critically depended on the chamber height and the relaxation timescale was slower for larger chamber heights. Thus, to probe non–steady state dynamics of the microchamber, we specifically used the air-immersion objective lens configuration for the rest of the experiments detailed here.

To study the effect of material properties of the superstrate (thermal conductivity, in particular) on temperature dynamics, we changed the superstrate of the microchambers from sapphire (κ_saph_ = 30 W/mK) to glass (κ_glass_ = 0.9 W/mK). Overall, the thermal conductivity of the superstrate had a minimal effect on the temperature dynamics for the chambers heights of 100 and 500 μm, and only showed a marginal effect when the chamber height was 300 μm (fig. S7).

To further understand the complex relationship between the chamber height and the temporal dynamics of temperature, we performed 2D numerical simulations using COMSOL Multiphysics for a fixed pump spot size of 10 μm (section S4). We calculated the temporal evolution of temperature in two extreme cases of the chamber height corresponding to 50 and 500 μm. Numerical simulations revealed that the time to reach steady state for a 500-μm chamber was about 17 min, while for a 50-μm chamber, it was about 7.5 min. However, the conclusions drawn here are limited to cases where natural air convection is the primary mechanism by which the microchamber interacts with the environment. Under these conditions, heat accumulates within the system and raises the global temperature.

We can conclude that the time to reach steady state, in the case of microchambers and closed systems in general imaged using air objectives, depends on the chamber height (translates to the volume of water) and is orders of magnitude higher than the expected theoretical value of 175 μs for a spot size of ∼10 μm. Hence, this case highlights the overall need to exercise caution when estimating the steady-state dynamics of the system, as well as the need to take into account the system as a whole, including its surroundings to obtain an accurate picture of the underlying thermodynamics. However, the conclusions drawn here apply to a closed microchambers with a finite height and cannot be extended to quasi-infinite systems as Δϕ_max_ diverges for an infinite system.

### Non–steady state thermometry: Planar heat source

**Fig. 4.**
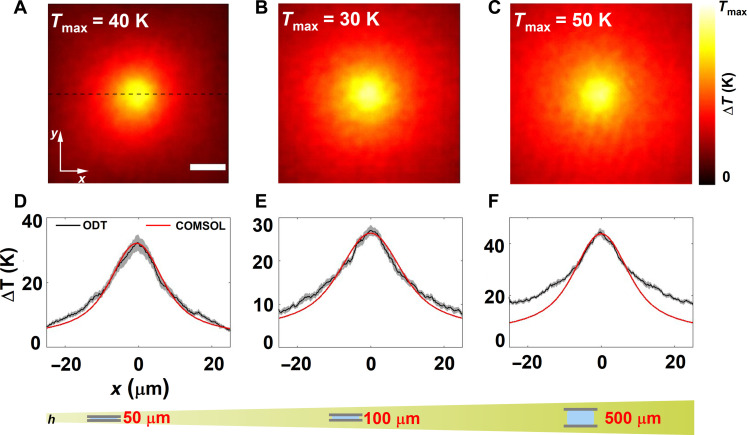
Application of ODT thermometry to planar heat sources. (**A** to **C**) Experimental thermal map at z = 0 measured using ODT for the chamber heights of 50, 100, and 500 μm, respectively. Scale bar: 10 µm. (**D** to **F**) Comparison of the line profile plotted across *y* = 0, shown as a dashed line, with the numerically calculated thermal profile. The pump duration was fixed to 80 ms and the camera frame rate to 10 Hz.

We first applied ODT thermometry to understand the thermal profiles of a planar heat source and systematically probed the microchambers in a pump-probe manner with three different chamber heights, keeping the beam size (12 μm) and pump-pulse duration (80 ms) constant. In these microchambers, similar to those used to measure the phase maps in Fig. 2, AuNRs were anchored to the glass substrate and thereby formed a planar heat source when excited by the pump laser Fig. 2. Figure 4 (A to C) shows the cross section of measured temperature profile (at *z* = 0) for chamber heights of 50, 100, and 500 μm, respectively. Numerical simulations using COMSOL were carried out to corroborate the experimental data. To compare the experimental data with simulations, we plotted the line profile along *y* = 0, represented as the dashed black line in Fig. 4A.

We found an excellent agreement between numerical simulations and experiments for chamber heights of 50 and 100 μm as shown by the line profiles in Fig. 4 (D and E). However, there is a mismatch for the 500-μm chamber, which we attributed to the slower relaxation dynamics. In detail, given that the measurements were performed in a pump-probe scheme, there is an intrinsic assumption that the system cools sufficiently fast enough such that the pump OFF state does not have any residual heat left from the previous heating cycle. For the 80-ms pump duration, this condition was not satisfied, as the pump duration was comparable to the frame time of the camera (*T*_pmp_ = 80 ms and *T*_cam_ = 100 ms). Hence, the residual heat in the system interfered with the measurement and appeared in the form of deviations from the theoretically expected $\frac{1}{r}$ profile. To verify this hypothesis, we probed the 500-μm chamber with different pump-pulse durations (5, 20, and 80 ms) while keeping the beam size constant. As expected, the line profiles extracted for the shorter pump-pulse duration of 5 and 20 ms matched well with the numerically calculated profiles (fig. S9).

To establish the non–steady state nature of the temperature dynamics, we benchmarked the ODT measurements against QPI thermometry. As mentioned earlier, QPI thermometry, in its most general form, assumes steady state and presupposes the $\frac{1}{r}$ decay in the temperature profile. The thermal profiles extracted using QPI match very well with that of ODT thermometry up to a DC shift due to the global heat accumulation as predicted by the temporal evolution of the phase gradient (Fig. 2 and fig. S10). As expected, the value of the constant shift between the thermal profiles extracted from QPI and ODT depends on the chamber height.

Temperature sensitivity was estimated by calculating the SD of the retrieved refractive index values in the background region (fig. S3). The SD was 1.21 × 10^−4^ refractive index units, which corresponds to a 0.7-K temperature change. This value determines the minimum distinguishable temperature value. To establish the reproducibility of the measurements, we repeated the measurement of the thermal profiles at the same spot using ODT in a microchamber 30 times, keeping the spot size and power constant. The temperature profiles are shown in Fig. 4 (D to F). Here, the bold line indicates the average of those replica measurements with the shaded regions representing the SD in each direction. Note a higher SD near the peak of the temperature increase of 2.7, 1.2, and 1.1 K for microchamber of heights 50, 100, and 500 μm, respectively. We attribute these larger fluctuations at the maximum temperature position to a combination of phase error in measurement, mechanical instabilities in the system, and homogeneity and anisotropy of AuNRs on the sample. Nevertheless, since this measurement is intrinsically shot noise–limited, the sensitivity can be further increased by increasing the number of holograms averaged per angle scan. Overall, by systematically studying the temperature profiles in microchambers with multiple chamber heights and pump durations and benchmarking the results with numerical simulations, we show that ODT thermometry can be applied to study a wide class of non–steady state thermodynamic systems.

### ODT thermometry: Nonplanar, spatially fixed heat sources

**Fig. 5.**
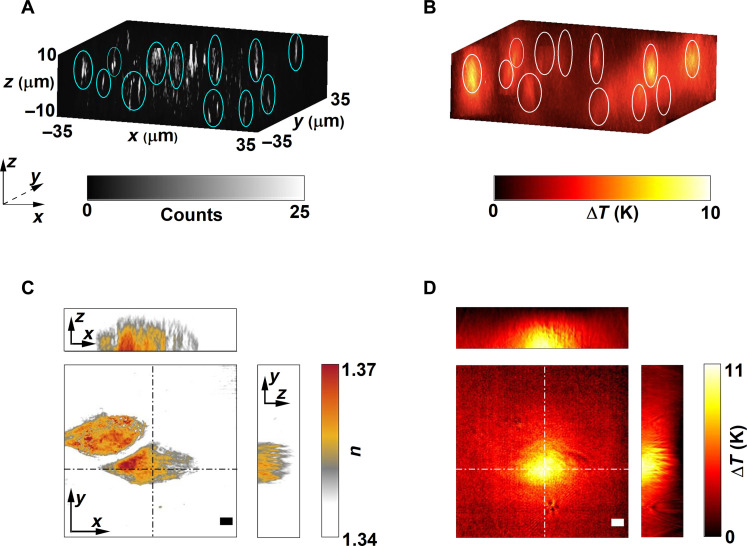
Nanothermometry with spatially fixed 3D heat sources. (**A** and **B**) Correlative scattering amplitude and 3D temperature maps of 150-nm AuNSs randomly dispersed within a polyacrylamide gel. (**C**) 3D refractive index map of a fixed A549 lung cancer cell that has ingested AuNRs. (**D**) Corresponding 3D temperature profile when the cell was pumped with a 785-nm laser. The circles act as a guide to the eye showing that the clusters are located in different planes. Scale bars: 5 µm.

So far, we have studied thermal maps of AuNRs anchored to the surface of a glass substrate acting as a planar source of heat. A unique advantage of ODT is that it can measure temperature profiles originating from 3D heat sources, for instance, from an ensemble of AuNSs of size 150 nm distributed in a 3D matrix (*39*). To validate the versatility of ODT to nonplanar heat sources and to demonstrate the method as a tool toward thermally characterizing a heterogeneous nanoparticle sample for potential mechanistic identification, we performed correlative QPI and wide-field ODT spatially resolved temperature measurements of 150-nm AuNSs embedded within polyacrylamide gel cast within microchambers with a height of 100 μm (Fig. 5, A and B). For these systems, numerical simulations are ill-suited and have limited predictive capability, as the particle distribution and composition are a-priori unknown. Figure 5A shows the reconstructed 3D intensity image of the AuNSs within the sample volume obtained through hologram propagation (*9*). The sample was then wide-field excited using the same pump laser, and Fig. 5B shows the resulting thermal maps. Figure 5B highlights the inherent property of ODT to colocalize the local temperature landscape with the 3D spatial distribution of complex objects. These data showcase the potential of the approach, the applicability to any resonantly excited nanoparticle system, and compatibility to perform correlative measurements. We further established the depth resolution of ODT by probing immobilized AuNRs in polyacrylamide gel by precisely moving the focus of the pump laser in the axial direction using a spatial light modulator (see section S6).

To further understand the applicability of ODT thermometry to systems with refractive index inhomogeneities, we probed an A549 lung cancer cell that had previously internalized AuNRs. We quantified the increase in local temperature caused by photothermal conversion from the AuNRs inside the cell upon resonant excitation (see Methods). Figure 5 (C and D) shows the 3D tomogram of a representative single cell, alongside the induced 3D thermal profile upon irradiation with a 50-ms pump pulse, respectively. Here, the cell-internalized AuNRs act as nonplanar heat sources embedded inside a complex refractive index environment, represented here by the cell. In this particular experiment, although the AuNRs were distributed throughout the volume of the cell, the much smaller illumination laser spot defined the size of the heat source. Note that the temperature profile is smooth even in the presence of refractive index inhomogeneities given by the cell, thus making ODT approach applicable to scenarios with complex refractive index environment.

### ODT thermometry: Nanorod colloids

Apart from spatially fixed heat sources (AuNRs anchored on a glass substrate or clusters dispersed in polyacrylamide gels/biological cells), we applied ODT thermometry to measure the temperature profile proceeding from dynamic environments. As a model system, we selected colloidal AuNR solutions. In detail, we prepared microchambers with a height of 100 μm filled with solutions of AuNRs of varying concentration and probed them using ODT thermometry. In systems where the sources of heat can freely diffuse, large-scale spontaneous migration due to the formed temperature gradient, termed thermophoresis (*40*), represents a major bottleneck in the retrieval of temperature . When the nanoparticles are heated, they move in response to the temperature gradient. However, the thermophoretic mobility depends on size and composition of the nanoparticles and the duration of heating (*11*, *41*–*43*). To avoid large-scale thermophoresis in the sample, we fixed the pump-pulse duration to 20 ms. We also tracked the phase shift induced in the probe beam across multiple pump cycles to ensure that there was a minimal change in the phase difference profile, thereby showing that the heat source density per pump pulse did not vary markedly (fig. S12).

**Fig. 6.**
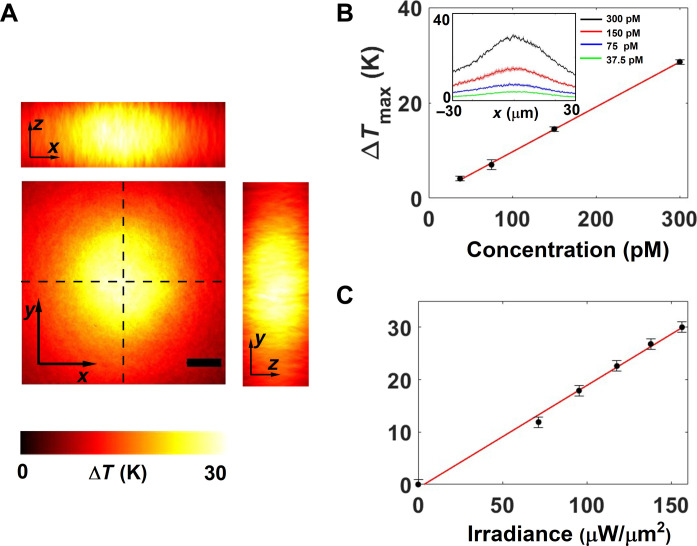
Nanothermometry of AuNR colloids. (**A**) 3D temperature of a colloidal sample of AuNRs in a microchamber with a height of 100 μm excited with a laser retrieved using ODT thermometry. The pump duration was kept at 20 ms, and the frame rate was at 10 Hz. Scale bar: 10 µm. (**B**) The maximum rise in temperature as a function of the concentration of AuNRs in the chamber. The input irradiance was fixed at 151 μW/μm^2^. Inset shows the line profiles of the temperature plotted along *y* = 0. (**C**) The maximum rise in temperature as a function of the input irradiance. The concentration of AuNRs was fixed at 300 pM. The error bars represent the SD of maximum temperature value for 10 experimental replicas.

Figure 6A shows the 3D thermal profile measured at AuNR concentration of 300 pM. To further characterize the temperature increase in colloidal nanoparticles, we systematically studied the temperature increase as a function of AuNR concentration and the input irradiance. Figure 6 (B and C) depicts the maximum temperature reached in the collodial system as a function of concentration and input irradiance, respectively. The linear dependence of the maximum temperature both on the concentration (at constant irradiance, 151 μW/μm^2^) and irradiance (at constant concentration, 300 pM) establishes the reliability of the temperature retrieval and shows that there is no large-scale thermophoresis due to localized heating and thermal gradient.

Together these three proof-of-principle experiments establish the power and advantage of ODT thermometry over other existing methodologies. Namely, ODT delivers 3D thermal profile distributions from complex nonplanar heat sources under transient thermodynamics regimes, i.e., not in steady state. However, these advantages come at the cost of relatively more complex experimental setup and data processing steps as well as longer acquisition times compared to other QPI thermometries.

## DISCUSSION

To summarize, this work delivers and validates an optical platform based on pump-probe ODT that can 3D map the temperature around nanoscopic heat sources in a precise, accurate, and potentially time-resolved manner. In addition, we have studied experimentally and numerically the thermodynamics of a model non–steady state system consisting of an optically heated microchamber. We unraveled an important relationship between the time to reach steady state and the chamber height and the thermal conductivity of the environment. We showed that ODT thermometry accurately retrieves the 3D temperature profile under non–steady state conditions and benchmarked these results against numerical simulations and QPI thermometry. We also showed the versatility of this technique by applying it to systems with nonplanar heat sources. We further presented a promising application by imaging the induced temperature profiles within biological cells upon plasmonic photothermal treatment. We also demonstrated its compatibility to retrieving temperatures from colloidal systems. All in all, the methods described in this work pave the way to providing unprecedented mechanistic insight at the single-particle level into nanoparticle systems involved in photocatalysis (*44*, *45*), photoemisson (*46*), thermionic emission (*47*), photoluminescence (*48*), photothermal therapy (*3*), thermal lensing (*7*), and microfluidic optical traps (*49*). We also anticipate that the conclusions drawn in the article will stimulate further experimental and theoretical investigations on the development of more accurate and faster thermometry techniques, which will represent a notable step forward to better understanding of heat-related processes at the nano and microscales.

## METHODS

### Sample preparation

AuNRs were synthesized using the method described in Nikoobakht and El-Sayed (*50*). The glass substrate was uniformly coated with the synthesized AuNRs using standard functionalization protocol described in detail in (*9*). The microchambers were prepared by placing silicon gaskets of predetermined thickness (50, 100, 300, and 500 μm) on the nanorod-coated glass substrate. The gap in the silicon gasket was filled with ∼10 μl of deionized water, and the chamber was closed with a sapphire superstrate. The inner diameter of the silicon gasket was about 8 mm in all the cases.

To immobilize nanoparticle clusters in polyacrylamide gels, we followed the standard operating protocol of preparing gels outlined by Bio-Rad. For nanothermometry experiments with biological cells, we used lung cancer cells (American Type Culture Collection, CCL-185) that had internalized AuNRs. The protocol for the sample preparation is as follows. Petri dishes were cleaned in 70% ethanol and sterilized in ultraviolet light for 10 to 20 min. We placed two silicon wells (gaskets) in a petri dish with a seeding concentration of about 5000 and 10,000 cells per well. Then, 500 μl of complete medium [Dulbecco’s modified Eagle medium + 10% fetal bovine serum (FBS) + 1% penicillin-streptomycin] was added to the cell droplet, and the cells were allowed to attach overnight. Then, the wells were washed with the medium without FBS. We added 4 ml of 2 nM AuNR solution to the wells, and they were allowed to incubate overnight. The wells were then washed again with medium without FBS. Later, the cells were washed twice with phosphate-buffered saline, incubated for 5 min in 4% paraformaldehyde (PFA) and for 15 min in 2% PFA, respectively, and kept in 1% PFA. The microchamber was prepared by removing 1% PFA and adding ∼10 μl of deionized water and closing the chamber with another glass substrate on the top and gently applying pressure to fix the silicon wells.

### Thermal imaging using ODT

ODT relies on calculating refractive index profile from multiple phase and amplitude images, measured by changing the angle of illumination, using the Fourier diffraction theorem (see section S2 for more details). Phase and amplitude images for different input angles were measured using an off-axis holographic microscope shown in section S1. The measured holograms were processed by first taking the Fourier transform, which revealed real, twin, and zero-order images in the *k*-space. The real image was filtered using a hard aperture selection, followed by frequency demodulation (*9*). The demodulated image was then inverse Fourier–transformed to get the complex electric field.

For retrieving thermal profiles, we measured multiple (*50*) complex electric field maps in a pump-probe manner for each given input angle. The resulting phase and amplitude maps were averaged to increase the signal-to-noise ratio and improve the phase sensitivity. Then, the scattered electric field *U*_s_(*x*, *y*) was calculated using the Rytov approximation as *U*_s_(*x*, *y*) = $\text{ln}\left(\frac{U\left(x,y\right)}{U_{\text{back}}\left(x,y\right)}\right)$ , where *U*(*x*, *y*) and *U*_back_(*x*, *y*) are the measured electric field maps with pump ON and pump OFF, respectively. This step was repeated for 20 different angles of incidence.

Last, the corresponding Ewald’s sphere was generated by combining all the scattered electric field maps, *U*_s_(*x*, *y*), in the Fourier domain according to the Fourier diffraction theorem (*32*). The inverse Fourier transform of the Ewald’s sphere results in the 3D refractive index image, which was then transformed into 3D thermal map using an empirical equation

$$n\left(T\right)=\sum_{j=0}^{P}‍b_{j}T^{j}$$ (1)

where *T* was the temperature and *b_j_* values are expansion coefficients. We considered up to *P* = 4 with values given as (*51*) *b*_0_ = 1.34359, *b*_1_ = −1.0514 × 10^−4^, *b*_2_ = −1.5692 × 10^−6^, *b*_3_ = 5.7538 × 10^−9^, and *b*_4_ = −1.2873 × 10^−11^.
