# Supplementary material for: Non–steady state thermometry with optical diffraction tomography
Source: Sci Adv. 2024 Mar 22;10(12):eadk5440. doi: 10.1126/sciadv.adk5440 (PMC10959403; doi:10.1126/sciadv.adk5440)
Supplement: Supplementary file 1 — Figs. S1 to S12 [file sciadv.adk5440_sm.pdf]

Science Advances
AAAS

Supplementary Materials for

Non–steady state thermometry with optical diffraction tomography

Adarsh B. Vasista *et al.*

Corresponding author: Jaime Ortega Arroyo, jarroyo@ethz.ch; Romain Quidant, rquidant@ethz.ch

Sci. Adv. **10**, eadk5440 (2024)
DOI: 10.1126/sciadv.adk5440

This PDF file includes:

Figs. S1 to S12

S1 Methods

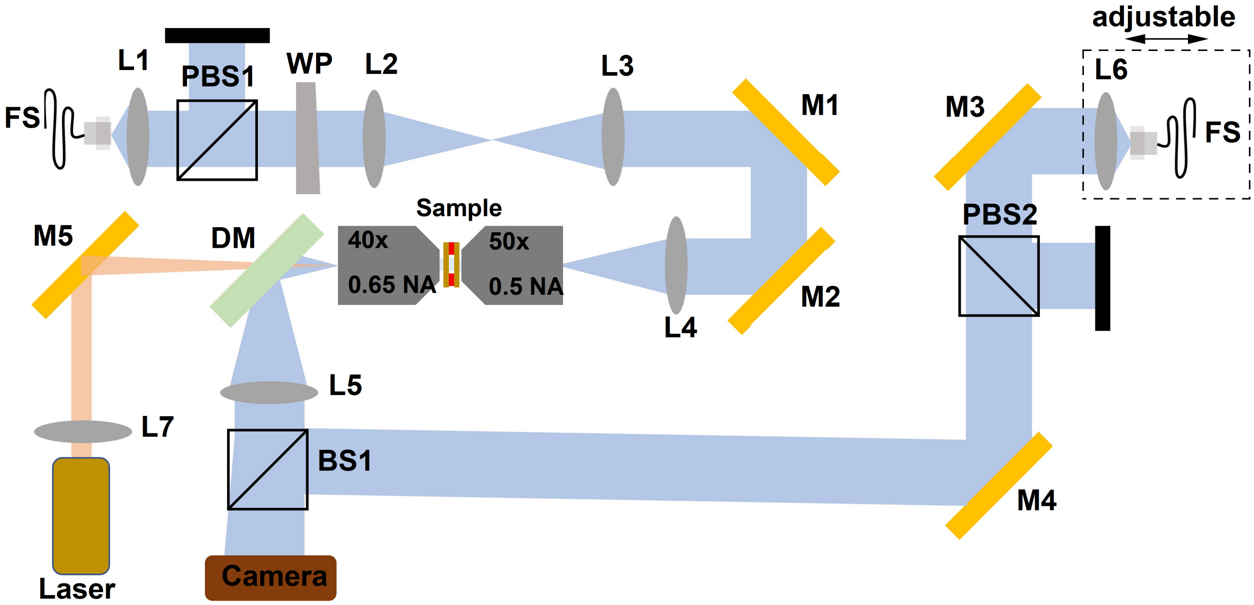

Figure S1: **Schematic of the pump-probe ODT thermometry setup**. L:Lens, M:mirror, PBS:Polarizing beam splitter, FS:Fiber source, BS:Beam splitter, DM:Dichroic mirror.

Figure S1 shows a schematic of the experimental setup used to measure phase maps in off-axis holography configuration in pump-probe manner. The probe laser (465 nm) was split into reference and object beams using a fiber splitter. In the object path, the probe laser was focused onto the back aperture of the objective lens (50x, 0.5NA) to generate a wide field illumination using a combination of lenses L2 (f_{L2}=30 mm), L3(f_{L3}=150 mm), and L4 (f_{L4}=150 mm). The probe light was collected in transmission configuration by a 40x 0.65 NA objective lens and the collected light was projected onto the camera using lens L5 (f_{L5}=250 mm) creating a magnification of 55 ($\frac{f_{L5}}{f_{objective}}$). The angle of illumination at the sample plane was controlled by the wedge prism WP. The reference beam was projected onto the camera at a small angle with respect to the optic axis of the microscope with the help of mirrors M3 and M4. The polarization of both object and reference path was fixed using polarizing beam splitters PBS1 and PBS2. The path length of the reference beam was adjusted by placing the fiber source module on an adjustable stage so as to match that of the object path. The gold nanorods were excited using a pump beam of wavelength 785 nm through 40x objective lens. The pump laser was not expanded and a long focal length lens, L5 (f_{L4}=500 mm), was used to focus the pump laser onto the back aperture of the objective lens to generate a spot size of ~10 μm. An FPGA card was used to synchronize the pump and probe lasers to the camera acquisition. The frame rate of the camera was set at 10Hz for all experiments and the pump and probe pulse duration was varied according to the requirements.

S2 Thermal imaging using optical diffraction tomography (ODT)

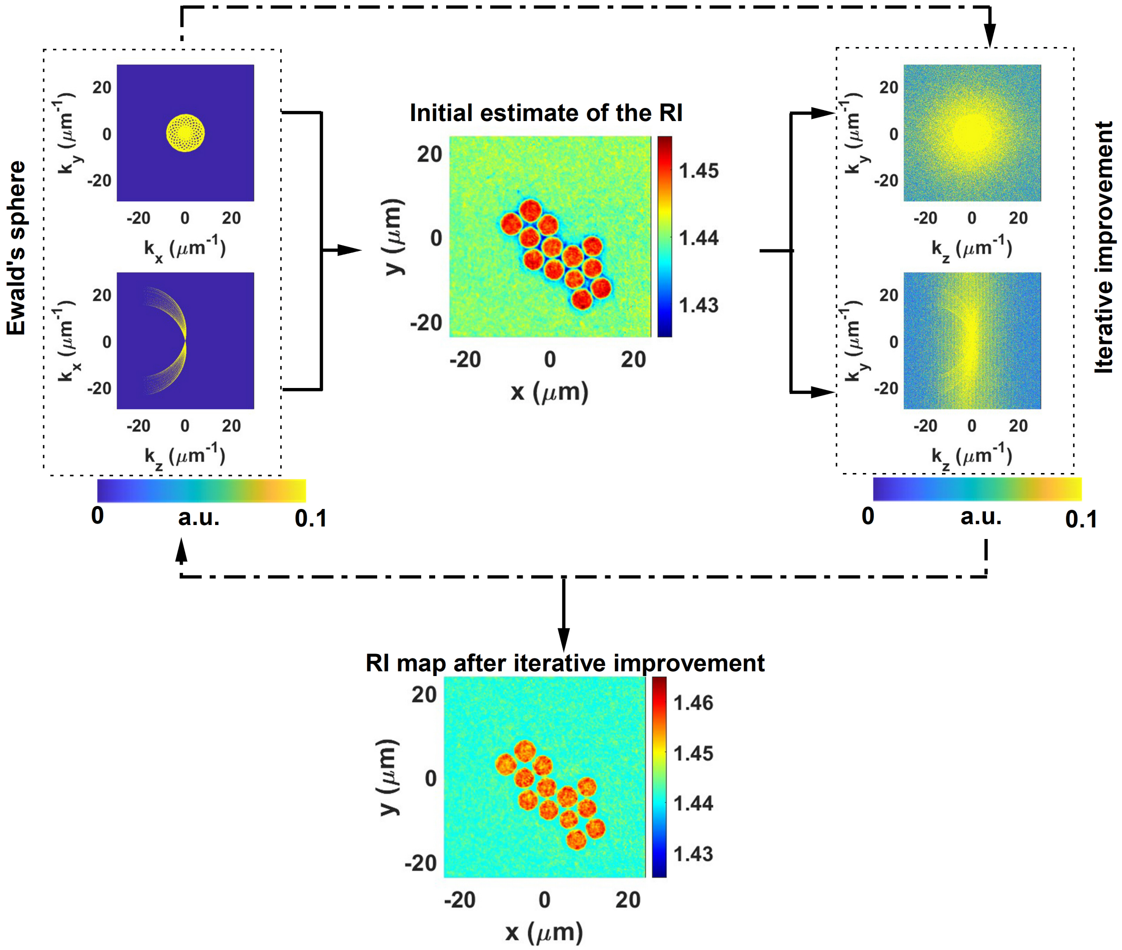

Figure S2: **Image processing pipeline for refractive index mapping.** Iterative algorithm to reconstruct the 3D refractive index profile of silica beads dispersed in PDMS matrix.

Optical diffraction tomography (ODT) has been widely used as an imaging tool to map the refractive index in 3D. ODT relies on calculating refractive index profile from multiple phase and amplitude images, measured by changing the angle of illumination, using the Fourier diffraction theorem. According to the said theorem, for any refractive index composition n(r) immersed in a medium of refractive index n_m we can write

$$\hat{F}(K_x, K_y, K_z) = \frac{ik_z}{\pi}\hat{U}_s(k_x, k_y; z = 0) \tag{S1}$$

where $\hat{F}$ is the 3D Fourier transform of the object function $f = -\frac{2\pi n_m}{\lambda^2}((\frac{n(r)}{n_m})^2 - 1)$ with λ as wavelength of illumination. $\hat{U}$ is the 2D Fourier transform of the scattering wave calculated using Rytov approximation. According to the Rytov approximation, we can express the scattering wave (U_s) as $U_s(x,y)=ln(\frac{U(x,y)}{U_{back}(x,y)})$ with $U(x,y)$ and $U_{back}(x,y)$ as the retrieved complex electric field in the presence of the sample and the background electric field respectively. In the case of ODT thermometry, $U(x,y)$ and $U_{back}(x,y)$ correspond to the complex electric fields measured with pump ON and pump OFF respectively. k_z is related to the lateral spatial frequencies (k_x, k_y) as $\sqrt{(\frac{2n_m\pi}{\lambda})^2 - k_x^2 - k_y^2}$. For each illumination angle the spatial frequencies of the incident wave vector changes and so does (K_x, K_y, K_z). Thus, one can map different regions of the k-space of the object function with measuring multiple 2D complex electric field images by changing the

angle of incidence. Thus mapped 3D object in the k-space is usually called as an *Ewald's sphere*. Finally, the inverse Fourier transform of $\hat{F}$ yields the 3D profile of the object function in real space which can readily be translated to the 3D refractive index profile.

It is important to note that due to the limited numerical aperture of the collection objective lens it is not possible to fill the whole *Ewald's sphere* through experimental data. This problem is referred to as the *missing cone* problem. The missing points on the *Ewald's sphere* were filled using iterative regularization procedure satisfying two physical constraints

- The refractive index of water at an elevated temperature can not be greater than that at an ambient temperature, as water has a negative thermo-optical co-effecient.
- The refractive index of the glass substrate is constant as the heat induced refractive index change in glass is negligible under experimental conditions.

Then the 3D refractive index maps were transformed to thermal maps using an empirical equation provided in the methods section of the main manuscript.

S2.1 Benchmarking ODT

As a control experiment we benchmarked the ODT technique by measuring refractive index of silica microparticles dispersed in PDMS matrix. In this case $U(x, y)$ was the complex electric field maps measured in the presence of the particle and $U_{back}(x, y)$ was the background electric field maps of bare PDMS matrix (without particles) (see equationS1). The initial estimation of refractive index of microparticles was improved using the iterative regularization step with constraint as: the refractive index of beads is greater than the refractive index of PDMS. Figure S2 shows the schematic of the tomographic reconstruction of the refractive index profile of silica microparticles dispersed in PDMS matrix and also indicates the effect of iterative regularization.

S2.2 Phase and temperature sensitivity

To further characterize the sensitivity of thermal imaging using ODT technique, we performed statistical analysis of the phase map as well as the retrieved refractive index map. The phase senstivity, which is the minimum distinguishable phase change, was calculated by plotting the histogram of values in the background area (inside a square defined along the corner of the image) of the phase map as shown in figures S3(a) and (b). The histogram values were fit to a gaussian distribution and the sensitivity, σ_{phase}, was found to be 6 mrad. In a typical RI tomogram we use 20 of such phase maps by changing the input angles as shown in Figure S3(c). The RI sensitivity was calculated in a similar manner as that of the phase and was found to be σ_{RI}=1.21x10^{-4} which corresponds to a temperature sensitivity of δT=0.7K (see figures S3 (d) and (e)).

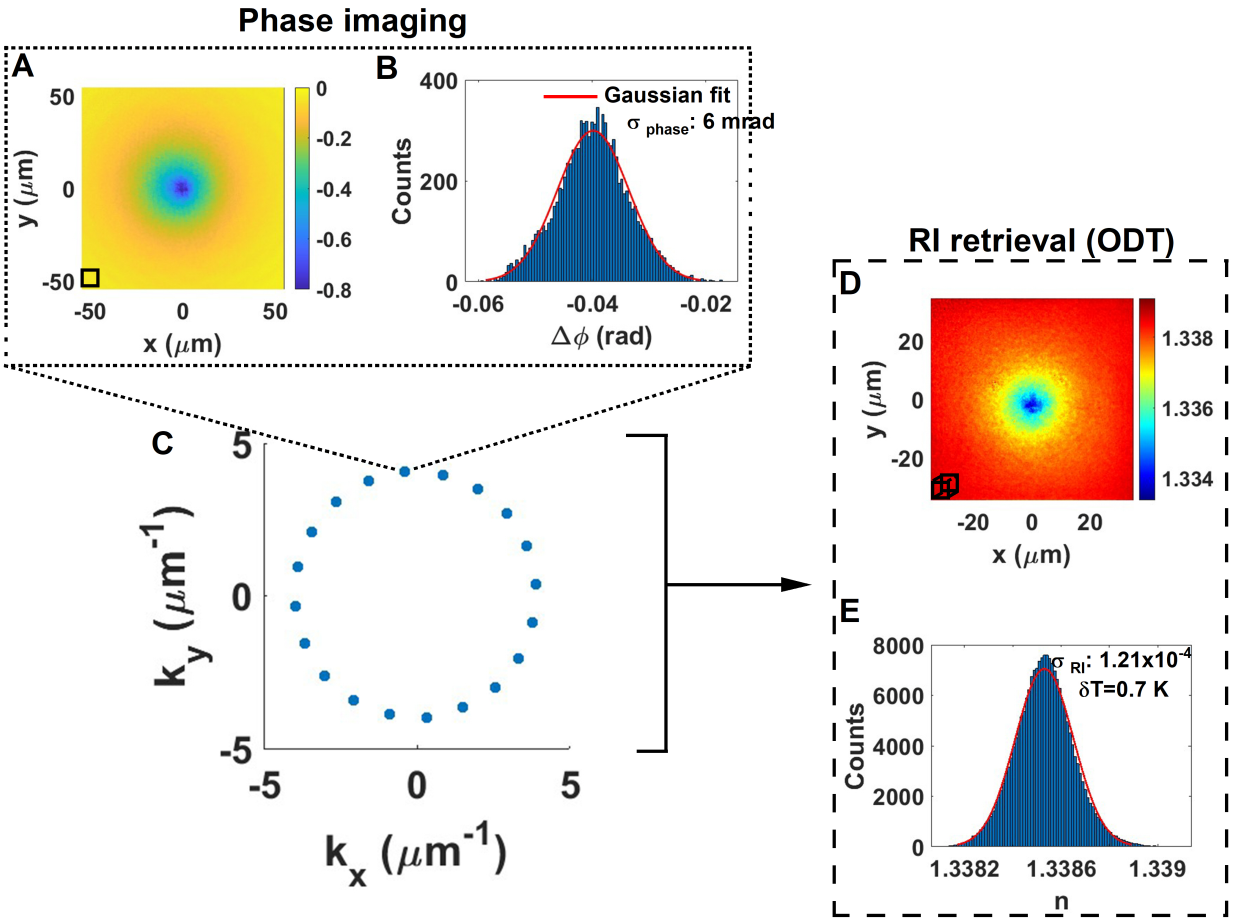

Figure S3: **Phase and temperature sensitivity analysis.** **(A)** A representative phase difference map measured for a single angle of incidence. **(B)** Distribution of background phase values inside the black square shown in (A). **(C)** Variation of (k_x,k_y) as a function of input angles. **(D)** Corresponding refractive index map. **(E)** Distribution of background refractive index values inside the black cube shown in (D).

S3 Thermal imaging using QPI

The algorithm to retrieve temperature map from an optical phase difference map has been outlined in detail in Ref.*27*. The measured phase difference map was converted into optical path difference (OPD) map. Then, the temperature retrieval was done in three steps:

1. We assume that the system has reached steady state distribution of the form $\frac{P_0}{r}$, where P_0 is the absorbed power and r is the radial coordinate. Then, we define the Green's function for OPD as $G_{OPD} = \beta_1 sinh^{-1}(\frac{h}{\sqrt{x^2+y^2}})$ where h is the height of the microchamber and β_1 corresponds to the first order thermo-optical co-efficient of water. We estimated the heat source density (HSD) by denconvolution of the OPD map and the G_{OPD} using Tikhonov deconvolution method.
2. We then convolved the obtained HSD map with the Green's function for Laplace equation, $G_{Th} = \frac{1}{4\pi\kappa_{water}r}$ where κ_{water} is the thermal conductivity of water, to get an initial estimate of temperature.
3. To consider the higher order thermo-optical co-effecients, we iteratively minimized the difference between the measured OPD map, l_0(x,y), and a path difference map calculated using $\int_0^h \delta n(x,y,z)dz$, where $\delta n(x,y,z)$ is the change in refractive index of water calculated using the retrieved temperature map by considering higher orders (up to 4) of thermo-optical co-effecients (see methods) .

For thermal imaging using QPI, we averaged twenty different phase maps measured by changing the angle of incidence in a manner similar to the building of *Ewald's sphere*, but in 2D. The resulting OPD map was employed in the temperature retrieval.

S3.1 Effect of chamber height on temperature retrieval

As explained in the previous subsection, the retrieval of the temperature map is based on the assumption that the temperature has reached a steady state and is of the form $\frac{P_0}{r}$. This is strictly true only in the case where the medium of heat transfer is quasi-infinite in nature. However when the height of the chamber is relatively small one has to take the thermal properties of the superstrate into account as discussed in Ref. *30*. However, it is tricky to obtain a closed form expression for HSD through deconvolution of the OPD maps if one considers the complete 3 layer model. The deviation from the quasi infinite model in temperature gets reflected in the iterative minimization of OPD error (step 3 in the temperature retrieval) as shown in Figure S4. The deviations of the reconstructed OPD map from the measured one decreases as one increases the chamber height from 50 μm to 500μm.

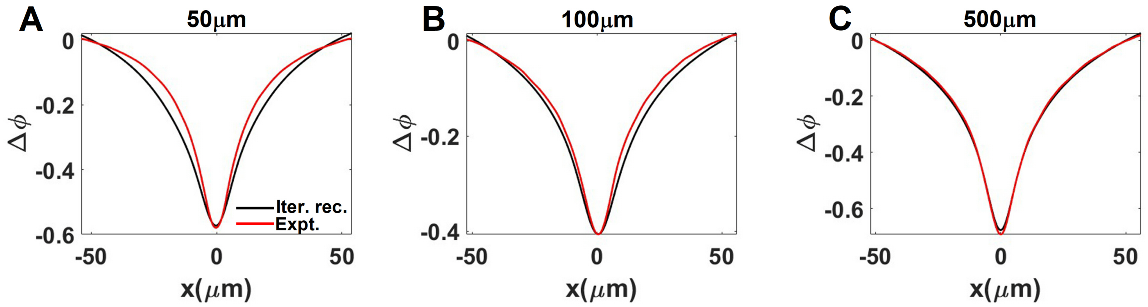

Figure S4: **Effect of chamber height on QPI-based thermometry**. **(A-C)** Phase difference line cuts (Y=0) for 50 μm (b) 100 μm (c) 500 μm microchambers.

S4 Temperature transients

Time taken to reach steady state for a quasi-infinite system is defined by its diffusivity, a_s, and the diameter of the heat source, D, and is of the form $\frac{D^2}{4a_s}$. Most of the experimental systems, eg. microchambers, are far from being quasi-infinite systems because of their limited chamber height. In such cases, the temperature dynamics depend crucially on the chamber height. To understand the temperature dynamics in microchambers we performed finite element method (FEM) based numerical simulations using COMSOL multiphysics. The lateral diameter of the chamber was fixed to 10 mm and the height of the chamber was varied. The substrate was defined as glass (κ_{glass}=0.9 W/mK) and the superstrate as sapphire (κ_{saph}=30W/mK). To mimic the experimental situation the chamber was heated by a gaussian heat source with a diameter of 10 μm. Natural air convection was assumed at the boundaries (both glass side and sapphire side) as in the case of experiments. Figure S5 shows numerically calculated evolution of maximum temperature as a function of pump duration for chamber heights of 50 μm and 500 μm. For both cases the time taken to reach steady state was significantly different from the values predicted by $\frac{D^2}{4a_s}$ i.e., 7.4 min for the chamber of height 50 μm and 16.7 min for the chamber of height 500 μm.

A striking feature to note is that the temperature reaches an intermediary plateau, a quasi steady state, before reaching the actual steady state. This can be understood by considering two thermalizing regimes of the system: *local* and *global*. When the ensemble of nanoparticles were heated in a thermodynamically closed system like a microchamber, the heated nanoparticles thermalize fast with its local environment and reaches an intermediary steady state which is termed as the *local* thermalization regime (the plateau in figure S5). The rate at which the entire microchamber thermalize is determined, primarily, by the natural convection at the boundaries. This *global* thermalization happened at a slower pace and at a higher temperature which

was a function of the volume of liquid inside the chamber. Such temperature evloution has been observed in the past in the case of multi nanoparticle heating

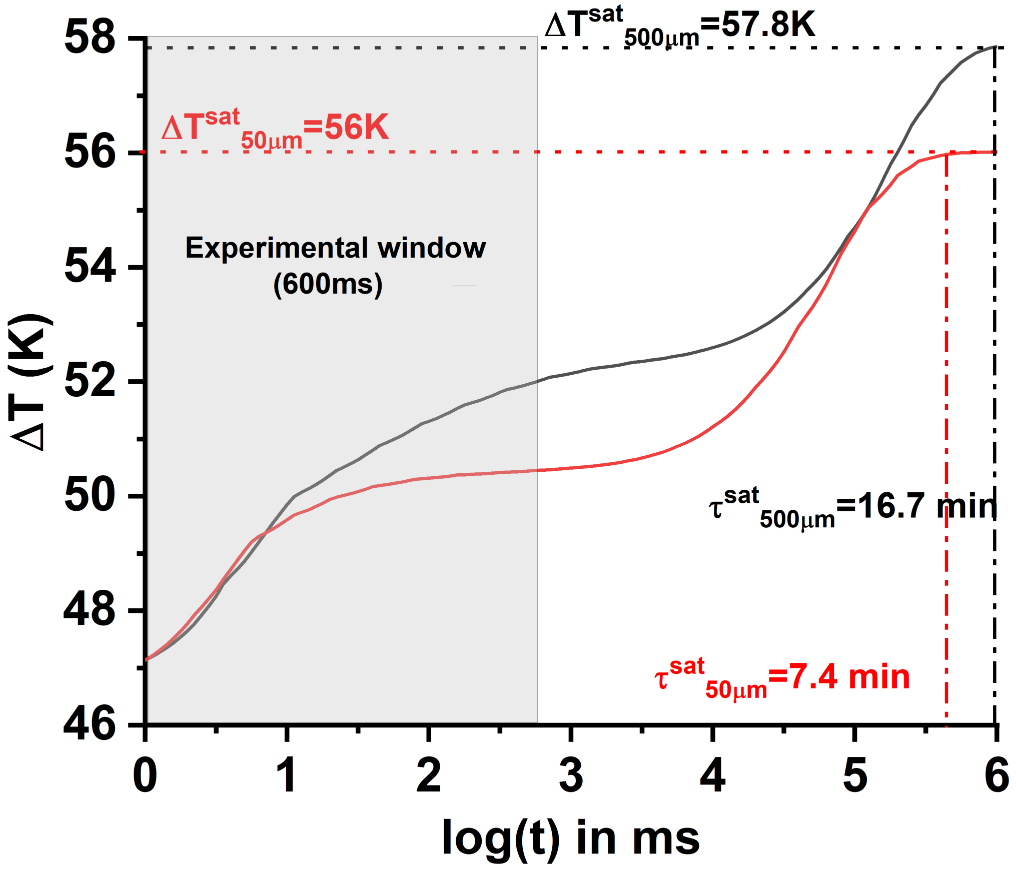

Figure S5: **Temporal evolution of the temperature gradient within a microchamber.** Numerically calculated thermal dynamics for microchamber of 500 μm and 50 μm for a fixed heat source size.

To understand the effect of substrate thickness on the temporal evolution of the thermal profile, we performed numerical calculations considering two different substrate thicknesses: 100 μm and 1 mm. Figure S6 shows the calculated thermal profiles of a microchamber of height 500 μm with 1 mm and 100 μm thick substrates respectively. The beam size was kept at 10 μm. The microchamber with thinner substrate reaches the steady state faster albeit at a higher temperature compared to a chamber with a thicker substrate. The increase in the time to reach steady state for the microchambers with thicker substrate was due to the increase in the volume of the material to thermalize compared to the chambers with the thin substrate. As the sources of heat were located in glass - water interface, the thicker substrate introduces a longer path in glass for the heat to diffuse and decay. This makes the effect of air convection (heat accumulation in the chamber) comparatively lesser than the thin substrate case.

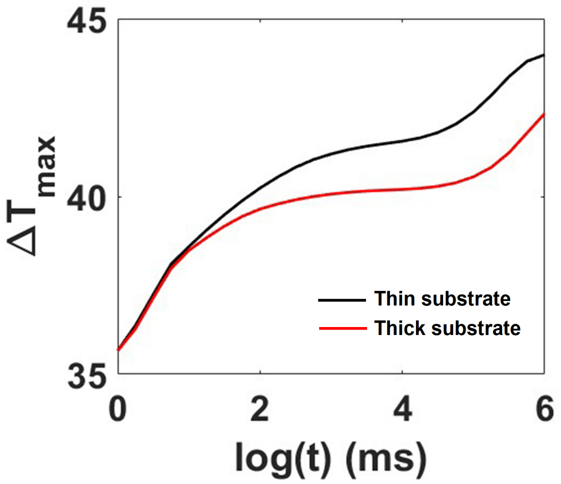

Figure S6: **Effect of substrate thickness of the temperature dynamics.** Numerically calculated time evolution of temperature profile for a microchamber of height 500 μm with thick substrate (1 mm) and thin substrate (100 μm).

S4.1 Effect of superstrate on temperature dynamics

To understand the effect of the thermal conductivity of the superstrate on temperature dynamics we probed microchambers with glass and sapphire as superstrates keeping glass as the substrate. Figure S7 shows the evolution of normalized phase accumulation ($\Delta\phi_{max}$) as a function of three different chamber heights. For both 100 μm and 500 μm chambers the phase evolution was almost independent of the superstrate while there was a marginal change for a 300 μm chamber. For the chamber height of 100 μm, the volume of liquid itself is small enough and introduction of sapphire as the superstrate does not quicken the temporal evolution of temperature, atleast within the experimental setting. Also, if the superstrate is very far (500 μm case) to the heat source the thermodynamics is mostly defined by the natural convection and thermal conductivity of the superstrate does not have a major role to play. However with an intermediary height (300 μm), sapphire plays a role in quickening the time evolution by acting as an efficient heat sink.

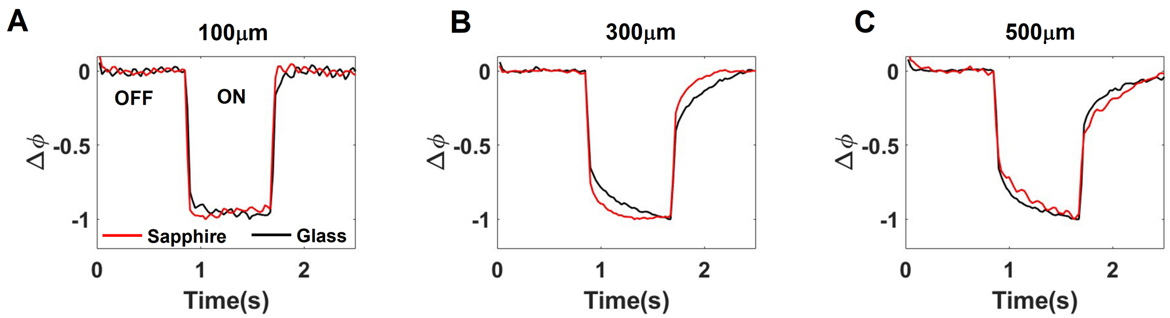

Figure S7: **Effect of superstrate thermal conductivity on the temperature dynamics.** Experimental phase-resolved measurements for chamber heights (a) 100 μm , (b) 300 μm, and (c) 500 μm.

S4.2 Effect of thermal conductivity of the surroundings on the temperature dynamics

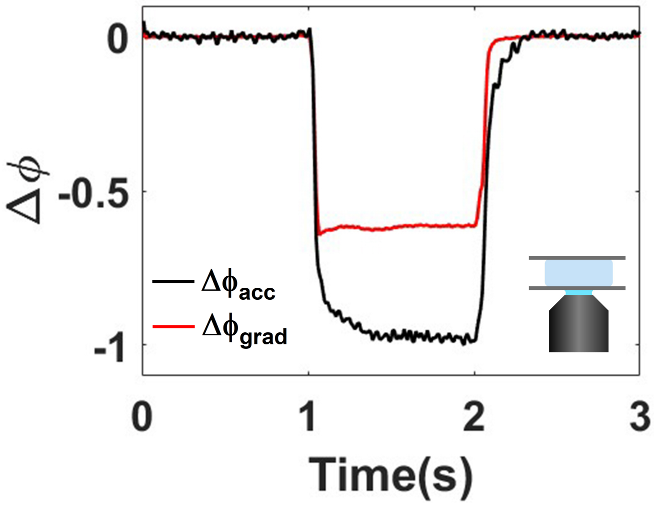

Figure S8: **Thermal conductivity of the surroundings affects the temperature dynamics.** Evolution of phase difference for a 500 μm chamber probed using an oil immersion objective lens.

Figure S8 shows the temporal evolution of phase gradient ($\Delta\phi_{grad}$) and maximum phase accumulation ($\Delta\phi_{acc}$) for a microchamber of height 500 μm when probed using an oil immersion objective lens for a pump duration of 1 s. The phase accumulation saturates within the time duration of the experiment while the phase gradient fluctuates before saturating (similar to figure 2).

S5 Non-steady state thermometry of planar heat sources with ODT

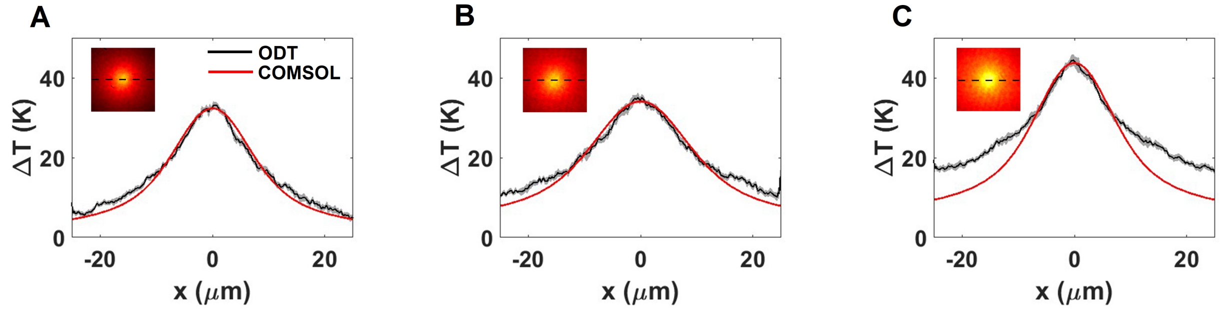

Figure S9: **Effect of pump pulse duration on ODT-thermometry**. Comparison of the line profile of the temperature along Y=0 for a chamber of height 500 μm pump duration (A) 5 ms, (B) 20 ms, and (C) 80 ms respectively with the numerically calculated thermal profile. Insets represent the XY crosscuts of the temperature profile in each case.

To better understand the deviation of the thermal profile of the microchamber of height 500 μm from the numerically calculated one, we probed the microchamber as a function of pump pulse durations. Figures S9 (a) - (c) show the comparison of line profiles of temperature as retrieved by ODT thermometry and numerical

calculations using COMSOL for a pump pulse duration of 5 ms, 20 ms, and 80 ms respectively. The camera frame rate was fixed at 10 Hz. The thermal profiles retrieved by ODT match very well with the numerical simulations for a pump pulse duration of 5 ms and 20 ms as there was minimal residual heat interference in the pump-probe cycle. However, in the case of an 80 ms pump pulse, there is a considerable interference of the residual heat resulting in the deviation of the thermal profile from the numerically calculated one.

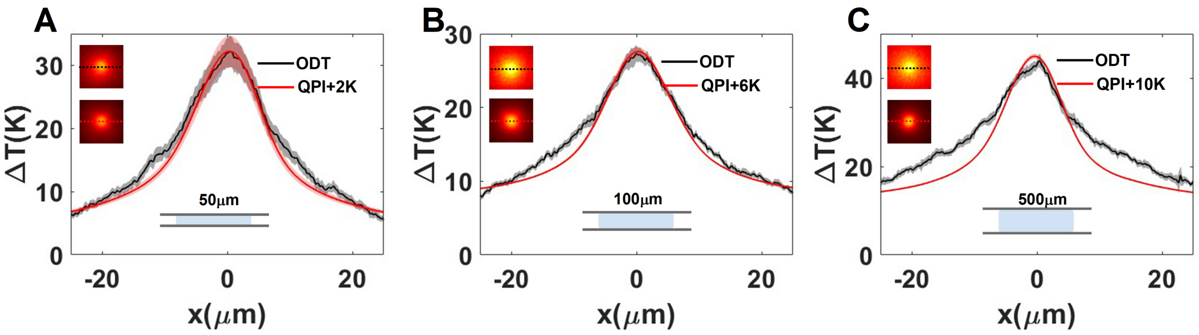

Figure S10: **Effect of chamber height of the temperature retrieved via ODT and QPI**. Comparison of the line profile of the temperature along Y=0 for chamber heights (a) 50 μm, (b) 100 μm , and (c) 500 μm respectively. The pump duration was kept at 80 ms and the camera rate at 10 Hz. Insets represent the XY crosscuts of the temperature profile in each case.

We utilized QPI thermometry to benchmark the thermal profiles retrieved by ODT technique. In the case of microchambers, due to the accumulation of heat, the system experiences a constant increase in the thermal floor keeping the shape of the thermal profile the same, thus pushing the system out of the steady state. As mentioned in the previous sections, QPI thermometry is based on two important assumptions: (i) The system has reached the steady state and the temperature profile follows a $\frac{1}{r}$ decay. (ii) The sources of heat are all located in one plane. Figures S10 (a)-(c) show the comparison of the line profile of the temperature along Y=0 for microchamber of height 50 μm, 100 μm, and 500 μm respectively. The pump pulse duration was kept at 80 ms and the camera frame rate at 10 Hz. A constant value was added to the temperature profiles retrieved by QPI for better visualization. The shape of the thermal profiles retrieved by ODT technique matched very well with QPI except for the constant value of the thermal floor, as predicted by the phase imaging, showing the microchamber was out of steady state.

S6 Depth resolution in ODT

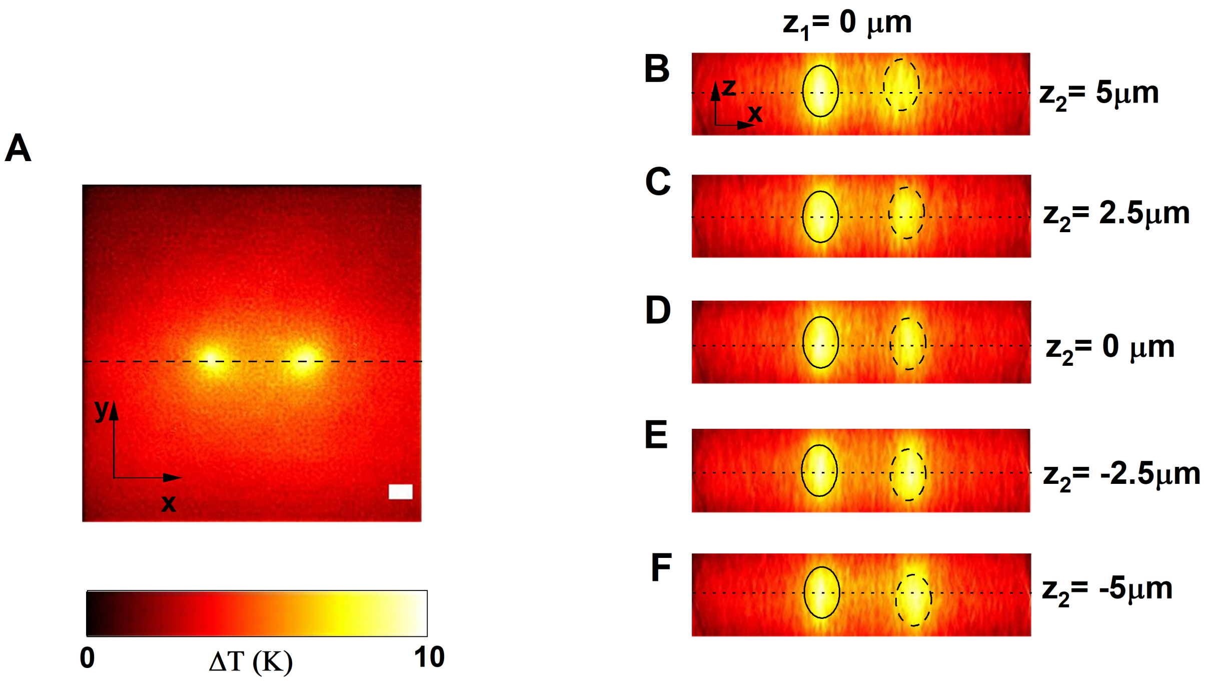

Figure S11: **Depth-resolved temperature measurements.** **(A)** Lateral cross section of thermal map of Au NRs homogeneously distributed in polyacrylamide gel and excited using two focused laser beams. Both laser beams are in focus (z_1=0, z_2=0). The scale bar is 5 μm. **(B - F)** Axial cross section of the thermal maps measured for foci of the laser beam 2 as z_2= 5, 2.5, 0, -2.5, 5 μm respectively. The focus of the laser beam 1 was fixed at z_1=0 μm. The circles act as a guide to the eye showing the location of maximum of temperature

To study the depth resolution of the ODT technique, we investigated a system where the temperature profile is known a-priori and can be systematically tuned by controlling the optical landscape. For this we used microchambers containing AuNRs distributed homogeneously inside hydrated polyacrylamide gels acting as a 3D heating source (see methods section of the main manuscript for the details on preparation of polyacrylamide gels). Due to the high density of AuNRs, upon resonant excitation the system behaves in the collective heating regime.

To control the thermal profile, we used a spatial light modulator (SLM) to produce two focused laser beams that were systematically displaced relative to each other. The axial displacement of the beam's foci induced by the SLM have been calibrated a priori to allow for sensitive changes of the beams position. In this case, the two laser spots act as sources of heat in multiple planes which can be individually moved. As we know how the spatial distribution of the heat sources interact with the beam, we can make accurate predictions of the resulting temperature profile. To test the depth of resolution, we spatially fixed one of the laser beams and axially moved the other one. Figure S11 shows the measured temperature maps for five

different axial positions of the laser spot. The spatial position of the temperature maximum follows the laser spot in the axial direction (see figure S11 b - f), clearly demonstrating the applicability of ODT to resolve heat sources distributed in different planes, even in the collective heating regime.

S7 ODT thermometry of Au nanorod colloids

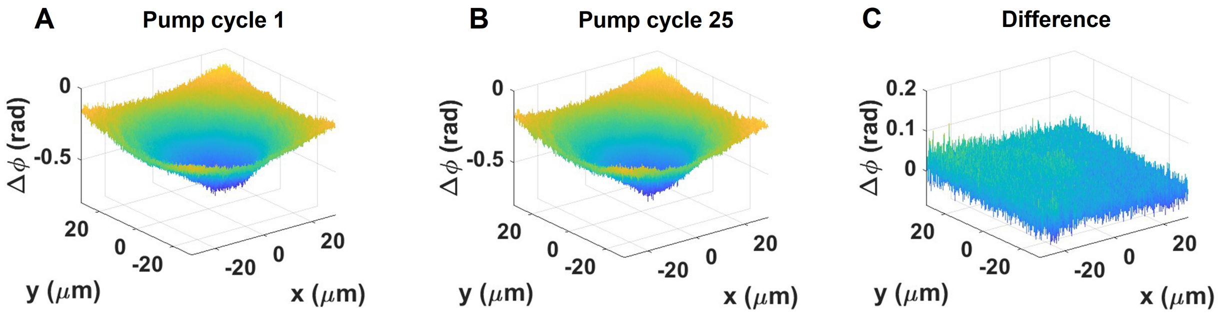

Figure S12: **Thermophoresis does not affect the temperature retrieval.** **(A,B)** Experimentally measured phase difference images for pump cycles 1 and 25 respectively. **(C)** The difference of the phase images for pump cycles 1 and 25.

The main concern in the thermometry of colloids is the large-scale movement of the nanoparticles due to the generated temperature gradient, termed thermophoresis. To avoid thermophoresis, we kept the pump duration to 20 ms. We further investigated the phase difference map for different pump cycles. The phase difference map gives an estimate of the heat source density generated per pump cycle. Figure S12 shows the measured phase difference map for pump cycles 1 and 25 as well as the calculated difference between them. Notwithstanding multiple pump-probe cycles, the phase difference maps did not alter considerably demonstrating that the heat source density generated per pump pulse remained the same.
